# Application of protein structure alignments to iterated hidden Markov model protocols for structure prediction

**DOI:** 10.1186/1471-2105-7-410

**Published:** 2006-09-14

**Authors:** Eric D Scheeff, Philip E Bourne

**Affiliations:** 1 San Diego Supercomputer Center, University of California, San Diego, 9500 Gilman Dr., La Jolla, CA 92093-0537, USA; 2 Department of Pharmacology, University of California, San Diego, 9500 Gilman Dr., La Jolla, CA 92093, USA; 3 Present address: Razavi-Newman Center for Bioinformatics, The Salk Institute for Biological Studies, 10010 North Torrey Pines Rd., La Jolla, CA 92037, USA

## Abstract

**Background:**

One of the most powerful methods for the prediction of protein structure from sequence information alone is the iterative construction of profile-type models. Because profiles are built from sequence alignments, the sequences included in the alignment and the method used to align them will be important to the sensitivity of the resulting profile. The inclusion of highly diverse sequences will presumably produce a more powerful profile, but distantly related sequences can be difficult to align accurately using only sequence information. Therefore, it would be expected that the use of protein structure alignments to improve the selection and alignment of diverse sequence homologs might yield improved profiles. However, the actual utility of such an approach has remained unclear.

**Results:**

We explored several iterative protocols for the generation of profile hidden Markov models. These protocols were tailored to allow the inclusion of protein structure alignments in the process, and were used for large-scale creation and benchmarking of structure alignment-enhanced models. We found that models using structure alignments did not provide an overall improvement over sequence-only models for superfamily-level structure predictions. However, the results also revealed that the structure alignment-enhanced models were complimentary to the sequence-only models, particularly at the edge of the "twilight zone". When the two sets of models were combined, they provided improved results over sequence-only models alone. In addition, we found that the beneficial effects of the structure alignment-enhanced models could not be realized if the structure-based alignments were replaced with sequence-based alignments. Our experiments with different iterative protocols for sequence-only models also suggested that simple protocol modifications were unable to yield equivalent improvements to those provided by the structure alignment-enhanced models. Finally, we found that models using structure alignments provided fold-level structure assignments that were superior to those produced by sequence-only models.

**Conclusion:**

When attempting to predict the structure of remote homologs, we advocate a combined approach in which both traditional models and models incorporating structure alignments are used.

## Background

The current stream of genome sequence data has lead to a bottleneck between DNA sequencing and elucidation of protein function [1]. Therefore, considerable effort has been expended to develop computational methods to suggest functions for putative genes [2]. In particular, many researchers have focused on attempting to predict the structure of unknown proteins using only their sequences [3,4]. The structure of a protein provides some of the richest information about its possible functions, as well as hints as to important residues and the location of functional sites [5-7].

Homology-based methods for structure prediction rely on the observation that proteins that share a common ancestor will usually have similar sequences, and that, in turn, similar protein sequences produce proteins with similar fold and (often) similar functions [8]. However, a given protein structure can be formed by a highly diverse array of possible sequences, and over long evolutionary time scales protein sequence divergence can be extensive [9,10]. Proteins which have similar structures may display such a paucity of sequence similarity that detection via current sequence homology search methodologies is not possible [11,12].

The profile-type methods address this problem by incorporating family-specific information inherent in multiple sequence alignments into sequence searches [13-15], and they can be generated automatically based on alignments built in an iterative fashion [16-18]. Hidden Markov models (HMMs) have been adapted for use as a particularly powerful profiling method [11,19,20], and have therefore been termed "profile hidden Markov models" [13]. As an alternative to iteration, profile HMMs can also be built based on carefully constructed seed alignments [21]. Profile HMMs built using either method can also be used as a panel of models, against which unknown sequences may be tested for similarity to a known family or superfamily [21,22].

Because profile HMMs model the information present in a sequence alignment, they are affected by quality of the input alignment [13], but the accuracy of alignments based only on sequence can be limited in cases of distant homology [23-25]. However, sequences at this level of similarity are the most informative sequences with which to build a model, as they will clearly demonstrate which sections of the family are well conserved, and the exact nature of the conservation.

Given the limitations inherent in sequence alignments as inputs for profile HMMs, researchers have explored the use of sequence alignments derived directly from structural alignments of proteins, particularly in cases where superfamily-level assignments are desired [22,26-31]. Structural alignments eliminate many of the problems with standard seed alignments. First, they can provide a highly diverse set of sequences from a variety of superfamily members, with more diversity than might be found even with an extensive iterative search. Second, because the structures are known, structure alignments can be used to provide an accurate alignment of the sequences.

One limitation in using structure alignments for profile HMMs is the weak level of structure representation in many superfamilies. In addition, coverage of a given superfamily is often strong in one area and weak in others. Hence, models made from alignments of these sequences, even if supplemented with additional homologous sequence, may not optimally describe the entire superfamily in question. It has also been suggested that there is an optimal range of sequence similarity for training of profile HMMs, and that using a very broad range of diverse sequence can lead to "profile dilution", reducing model quality [29,32].

Accordingly, the literature record for the use of structure alignments for profile HMMs yields mixed results. Several researchers, using a variety of experimental arrangements, have reported that HMMs based on structure alignments do not provide a benefit over standard HMMs. Gough *et al*. and Sillitoe *et al*. have suggested that pools of models, each built iteratively from a single structural representative (referred to here as the "master" sequence) provide better performance at the superfamily level than models incorporating structure alignments of multiple masters [22,30]. Sillitoe *et al*. also reported that combining the two types of models yielded essentially no benefit [30]. Griffiths-Jones and Bateman compared HMMs built from seed alignments based on structure against HMMs built from seed alignments based only on sequence information, and concluded that there was no benefit to structure alignments for the production of profile HMMs [27]. However, their analysis was done for family-level sequence targets, not the superfamily level targets that represent a more difficult challenge for homology search. In addition, they did not assess the value of structural alignments to the augmentation of iterative alignment procedures, as we do here.

Other researchers have provided qualified support for the use of structure alignments in the construction of profiles (usually using PSI-BLAST [16,17] instead of HMMs). Panchenko and Bryant reported a small improvement in structure prediction accuracy when profile seed alignments were created using structure-based, rather than sequence-based, alignments [29]. However, though their method used PSI-BLAST, their protocol did not include any iteration. Kelley *et al*. used structure alignments to augment iterative profile construction, and reported improved results when attempting to predict very distant homologous relationships. However, some of the improvement reported was based on the combination of the structure alignments with additional structure-derived information (secondary structure and solvent accessibility) [28]. Other groups have tested structure alignments when applied to newer profile-to-profile approaches, as opposed to the usual profile-to-sequence approach. Tang *et al*. explored the inclusion of structure alignments in the construction of profiles in a profile-to-profile approach based on PSI-BLAST [31]. Similar to the findings of Kelly *et al*., they found that profiles utilizing structure alignments provided some improvement over standard models, and that this increase in performance could be furthered through the addition of other structural data into the models. Casbon and Saqi tested a profile-to-profile approach relying on a hybrid PSI-BLAST/HMM protocol [26]. They found that the structure-alignment models had similar performance to the standard models at a low error rate, and weaker performance at a higher error rate. However, they noted that in some superfamilies the structure-alignment based models had a clear advantage over the standard models, and *vice versa*. This suggests that the two types of models might be complementary if used in combined searches.

We undertook a large-scale assessment of the utility of structure alignments for the generation of profile HMMs, using the traditional sequence-to-profile method. First, we tested several iterative protocols to determine which method generated the most sensitive profile HMMs with HMMER [13], using only sequence information. The aim was the generation of models that represent the sequence space around a single structural domain representative (the "single-master" models). Next, we developed a protocol for the production of merged sequence alignments, built by combining the sequence alignments from several domains together based on a multiple structure alignment. The method is designed such that the best aspects of iterative sequence search and structural seed alignments are combined. A maximum amount of sequence information is gathered for each superfamily through iterative search, but a structural seed alignment is used to combine the information accurately. Finally, the combined superfamily alignments were used to train another profile HMM with HMMER. We term such models based on combined alignments "structure-linked alignment hidden Markov models", or SLAHMMs (pronounced "slams"). We show that SLAHMMs provide an improvement upon sequence-only models built though iteration, when they are used together in a combined search. Our study supports the notion that structural information, in the form of structure-based alignments, provides a useful enhancement to standard profile HMM models.

## Results and discussion

### Iterative strategy has only a small effect on model performance

In order to determine the best iterative methodology for building both single-master HMMs and SLAHMMs with HMMER, performance was compared for four different parameter sets (PS1-PS4, see methods for details). In all cases, an alignment was built through repetitive HMM searches against a sequence database (with re-training of the HMM after each cycle), but the parameter sets tested different cutoffs for sequence inclusion into the growing alignment, use of heuristics to improve the alignment, and the method for aligning sequences to the model to create the alignment. Test sequences (or "probes") were then searched against the resulting models and the correct or incorrect structure assignments recorded. The superfamily assignments provided in SCOP [33,34] were used as a standard of truth for purposes of benchmarking the methods (with modifications in a few cases, see methods).

**Figure 1:**
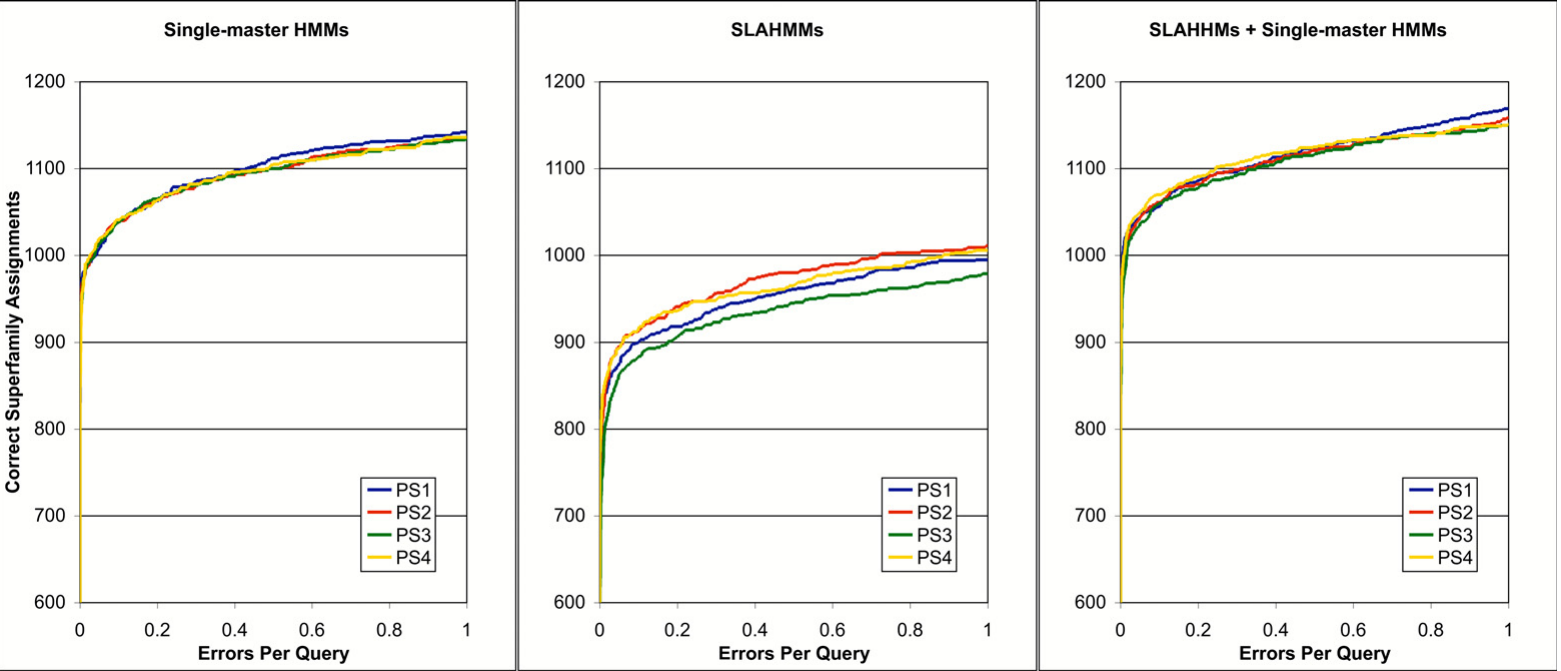
Relative performance of single-master HMMs, SLAHMMs, and the combined models with differing iteration parameter sets (PS), presented as a coverage vs. theoretical errors per query (EPQ) plot. The different parameter sets are defined in Table 2 and explained in the text. Values for correct assignments are truncated at 600 in order to emphasize differences between the various methods (no method had an error below 600 correct assignments).

When single-master HMMs were compared to each other for all four parameter sets (PS1-PS4), the difference in performance was extremely small (Figure 1). There was a slight advantage to a full heuristics methodology (PS1), which achieved more correct matches at a higher theoretical error per query (EPQ) level. However, this result indicates that traditional HMMs are surprisingly insensitive to the methods used to select and align sequences to the growing model.

Creation of SLAHMMs based on the same sequence alignments (combined based on structure alignments, see methods) also resulted in relatively similar performance between the various iterative parameters (Figure 1). However, in this case there was a larger variability in performance, suggesting that SLAHMMs are more sensitive to the methodology used to create the sequence alignment inputs. The parameter sets that repetitively re-aligned all sequences to the model (PS2 and PS4) performed better than those that did not (PS1 and PS3). This result suggests that sequence alignment drift does not pose a severe threat to the quality of SLAHMMs, and that re-aligning all sequences to the model may help to sharpen sequence patterns in a way that improves the resulting HMM.

However, the results also suggest that the use of the heuristics (to select sequences for addition to the model) applied in PS1 and PS2 had a beneficial effect on the quality of the models. Both PS1 and PS2 outperformed counterparts that did not use the heuristics. In addition to the small performance improvement, the heuristics provided the practical benefit of smaller alignment sizes (because they are more selective with respect to added sequences), and therefore lower computational overhead.

It was possible to use both sets of models together in a combined search, simply by placing both in a single database and searching the probes against this database. When performance of the combined models was compared for each parameter set, the difference again narrowed, much as it did for the single-master HMMs (Figure 1). Although the coverage vs. error curves were similar, PS1 was alone in identifying additional homologs at an EPQ of 1. The practical benefits of the heuristics used in PS1 and PS2, coupled with the slight performance benefits seen in the SLAHMM and combined tests, argued for the use of these heuristics in the generation of future models. Because of these factors, we chose to use PS1 for the remainder of the analysis.

### Single-master HMMs outperform SLAHMMs upon direct comparison

**Figure 2:**
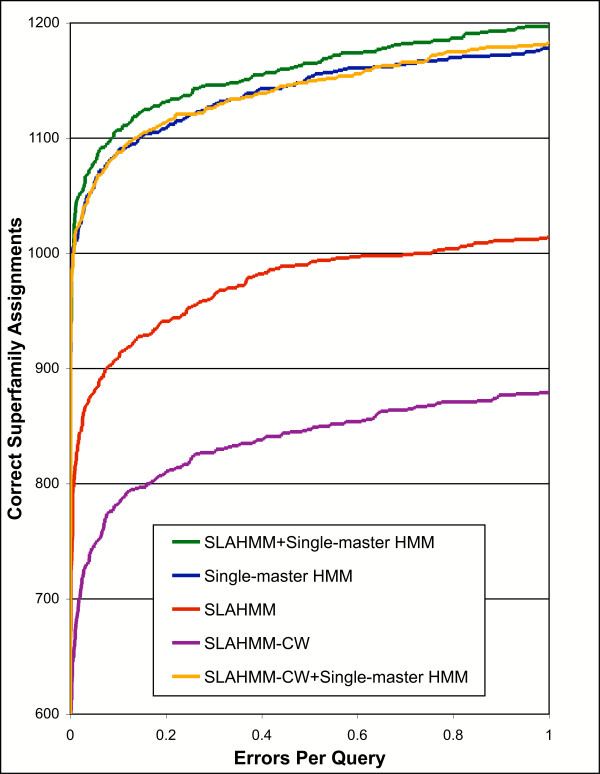
Relative performance of different types of HMMs in assignment of structure to sequence probes, presented as a coverage vs. error plot. SLAHMM-CW refers to models built in the same way as SLAHMMs, but using only sequence information to align the SCOP domains rather than a structural alignment (see text). Iterative parameters used for construction of all models were from PS1 (Table 2). Values for correct assignments are truncated at 600 in order to emphasize differences between the various methods (no method other than SLAHMM-CW had errors below 600 correct assignments).

When directly compared, single-master HMMs clearly outperformed SLAHMMs (Figure 2). This result is not surprising, as SLAHMMs attempt to incorporate all information about the superfamily in one model. In some cases, this may force the model away from sequences that would be easily annotated by a standard single-master HMM (i.e. SLAHMMs are designed to capture the most distant relationships, and therefore may miss easier ones).

**Figure 3:**
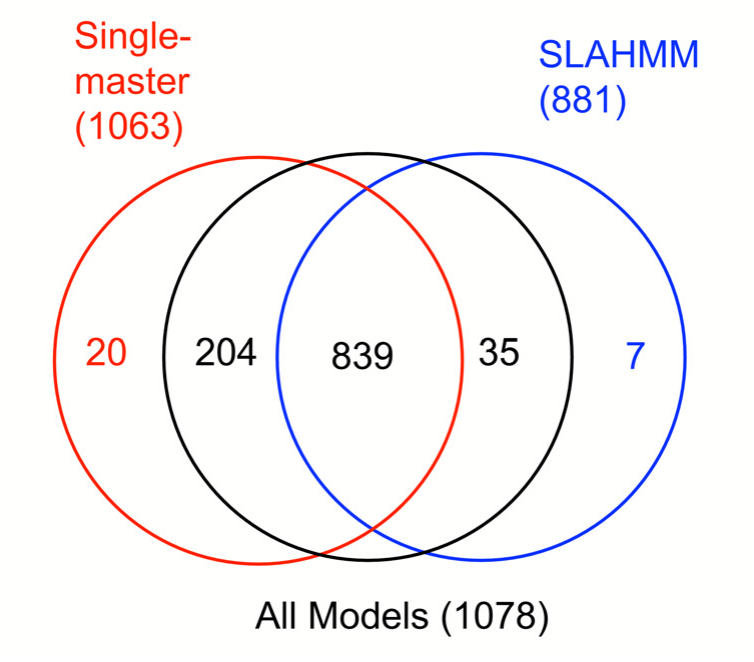
Venn diagram describing coverage overlap of the three primary model sets from PS1, when using a strict cutoff of 80 incorrect assignments (theoretical EPQ ~0.05). The numbers shown in parentheses near each model type designation refer to the total number of correct matches made by that model type prior to the cutoff point. Identical matches of the same probes by some or all of the three different methods are provided by the numbers in the set diagram. The completely unique matches by single-master HMMs and SLAHMMs are color coded to match the circle for that model type. "All Models" denotes the assignments made by the combined database of SLAHMMs and single-master HMMs used together in a single search.

Assessment of the specific probe assignments made by SLAHMMs vs. single-master HMMs confirmed that SLAHMMs made unique assignments. At a strict cutoff of 80 incorrect assignments (EPQ ~0.05), the two types of HMMs shared 839 correct assignments of the same probes (out of a total number of 1575 possible correct assignments, see methods). However, while single-master HMMs correctly assigned an additional set of 224 probes uniquely, SLAHMMs also correctly assigned 42 probes uniquely (Figure 3). The unique assignments made by SLAHMMs are summarized in Table 1; they come from a variety of superfamilies from all four major SCOP structural classes, indicating that the results are not simply the result of a few atypical superfamilies.

**Table 1:** Probes correctly assigned with SLAHMMs that were not assigned with single-master HMMs, using a strict cutoff of 80 incorrect assignments (theoretical EPQ ~0.05).

**Probe SCOP ID**	**E-value**	**SCOP Superfamily**	**Incorrect Assignments**	**ND1: (EPQ = 1)**	**ND2: nr60 (EPQ ~0.05)**	**ND3: nr60 (EPQ = 1)**
g1dy9.1	0.00018	b.47.1	0		X	

d1k6wa2	0.00021	c.1.9	0		X	

d1buoa_	0.00022	d.42.1	0			

d1dm9a_	0.00039	d.66.1	0		X	

d1rpxa_	0.00046	c.1.2	0			

d1hq3d_	0.00067	a.22.1	0			

d1at0__	0.00069	b.86.1	0			

d1hq3b_	0.0011	a.22.1	0			

d1kjqa2	0.0015	c.30.1	0			

d7taa_1	0.0016	b.71.1	0		X	

d2hrva_	0.0042	b.47.1	2	X	X	X

d1jfib_	0.0055	a.22.1	2		X	

d1hq3a_	0.0057	a.22.1	2			

d1bd0a2	0.0069	c.1.6	2		X	

d1hx0a1	0.0096	b.71.1	2		X	

d1bkra_	0.01	a.40.1	2			

d1bd3a_	0.012	c.61.1	3			

d1a0p_1	0.013	a.60.9	3			

d1afra_	0.014	a.25.1	5		X	

d1i6la_	0.016	c.26.1	6	X	X	X

d1k92a1	0.02	c.26.2	6	X	X	

d1dfaa2	0.026	d.95.2	8	X	X	

d1m4va1	0.031	b.40.2	8		X	

d1gsoa2	0.036	c.30.1	8	X	X	X

d1j8ca_	0.04	d.15.1	8	X		

d1dfca1	0.041	b.42.5	8	X	X	

d1dxea_	0.041	c.1.12	8		X	

d1efva2	0.048	c.31.1	9		X	

d1k3sa_	0.063	d.198.1	13		X	

d2pola2	0.066	d.131.1	14	X	X	

d2pola3	0.071	d.131.1	15			

d1k8kf_	0.1	d.198.2	24	X	X	X

d1efva1	0.11	c.29.1	24	X	X	

d1j9qa2	0.13	b.6.1	29			

d1gkpa1	0.14	b.92.1	36			

d2a0b__	0.19	a.24.10	41			

d1qo0d_	0.2	c.23.1	43		X	

d1hava_	0.21	b.47.1	45		X	

d1qg8a_	0.24	c.68.1	54			

d1hq3c_	0.25	a.22.1	56			

d1al3__	0.27	c.94.1	65	X	X	X

d1es9a_	0.28	c.23.10	68			

The "incorrect assignments" column refers to the number of incorrect assignments made at the point where the probe was correctly assigned a structure by a SLAHMM. The final three columns highlight the probes (marked with "X") that were not assigned by single-master HMMs even when they were allowed considerable advantages (the incorrect assignments column does not apply to these columns). ND1 ("Not Detected" 1) indicates the probes that could not be detected with a generous cutoff of up to 1575 incorrect assignments (theoretical EPQ = 1). ND2 and ND3 present results where the single-master HMMs were allowed to search the entire nr60 database for additional homologs during the training phase (see text for details).

The unique assignments made by SLAHMMs occur at high E-values, near the threshold at which errors start to be made. Indeed, only 10 unique assignments (~24%) were made prior to the first incorrect assignment. The rest were made after errors had been recorded. In contrast, of the unique assignments made by the single-master HMMs, 91 (~41%) were made prior to the first recorded error. This suggests that, as expected, SLAHMMs are very generalized models that capture more difficult assignments made right at the edge of "the twilight zone", but can miss easier matches.

If the single master HMMs are allowed a looser cutoff of up to 1575 errors (a theoretical EPQ of 1), they will correctly assign 31 of the 42 sequences that SLAHMMs capture at a cutoff of 80 (Table 1). Thus, part of the benefit provided by SLAHMMs is produced by simply improving the scores of probes that could be assigned, albeit with much lower confidence, using single-master HMMs. However, 11 structures are still not correctly assigned by single-master HMMs, even at this less-stringent cutoff (Table 1, column ND1). Of course, if SLAHMMs are also allowed a looser cutoff of 1575 errors, they will correctly assign additional structures that single-master HMMs miss at the same threshold. Indeed, SLAHMMs correctly assign 35 structures missed by single-master HMMs if both are held to a threshold of an EPQ of 1 (single-master HMMs make 198 unique hits at this cutoff).

### Combined models outperform single-master HMMs alone

The differing behaviors of the two types of models suggested that they would behave synergistically when combined, with single-master HMMs capturing targets that were easier, and SLAHMMs capturing targets that were more challenging. When the combined models were tested, the SLAHMMs were able to provide increased assignment performance, improving upon the capability of single-master HMMs (Figure 2). The combined models made 35 additional correct assignments at an EPQ of ~0.05, an improvement of ~3.3% (Figure 3). This pattern held throughout the sampled cutoffs, with 31 additional correct matches at an EPQ of 1.

Although the improvement provided by the addition of SLAHMMs appears modest, the structure of the experiment was likely to make the degree of improvement appear substantially smaller than actually realized in cases of very distant sequence homology. Our test set of probes was filtered such that all were non-trivial to recognize with BLAST [17] (see methods), but this does not mean that all of the probe assignments will be challenging ones, given the power of profile methodology [11]. We did not attempt to remove these easier to assign domains, because this may reduce the overall coverage of sequence space by the resulting model pool [30]. Therefore, it is expected that there will be a large "floor" of domains that are relatively easy to assign, which is consistent with the large degree of overlap between the results for single-master HMMs and SLAHMMs.

Interestingly, the single-master HMMs are able to make 20 assignments not made by the combined models at an EPQ of ~0.05 (Figure 3), reducing the net improvement to only 15 additional assignments (~1.4%). These assignments are missed because additional errors are also incurred by having both sets of models present, as a result of the added "noise". Such noise would be reduced in real-world use of SLAHMMs. The requirement for a set of test models with a sequence (and its accumulated hits) removed meant that a SLAHMM had to be made for each probe, yielding 1,575 models (see methods). These additional models doubled the size of the overall model pool, which presented a greater availability for random hits in a practical sense, especially at high E-values, where SLAHMMs seemed to provide the most benefit. However, in real-world use, only one model would be required per superfamily (242 models using the test set for this experiment), which would only produce a small relative increase in the number of models in the model pool.

### SLAHMMs require structural alignments

We sought to test the notion that structural alignments were truly essential for the production of SLAHMMs. It was possible that SLAHMMs simply benefited from a wider sampling of sequence based on structural information (because they combined the HMM search results of several distantly related sequences) but did not actually require the explicit incorporation of structural information in the form of a structure-based alignment. Further, it has been suggested that highly accurate alignments are not essential to the production of useful profile HMMs [27].

To see if structural alignments were required, we aligned our test protein domain sequences with ClustalW [35]. These sequence based-alignments were then used to build a unified superfamily alignment and SLAHMMs, in an otherwise identical fashion to the standard method. The results demonstrate that the benefits of SLAHMMs cannot be realized without the use of structural alignments (Figure 2). SLAHMMs built using a ClustalW alignment substantially underperform those built with a genuine structural alignment. Similarly, the ClustalW SLAHMMs do not provide any net improvement when combined with standard sequence-only models. At least in the case of SLAHMMs, or results indicate that highly accurate alignments are essential to the production of quality profile HMMs.

### SLAHMMs provide both practical and theoretical benefits

To make the iterative HMM searches computationally tractable during initial model building, BLAST was used to pre-filter a large pool of possible homologs from the sequence database for each SCOP master domain (see methods). Though it provides practical benefits, the BLAST pre-filter could also limit the possible sequence space available for training each single-master HMM. Thus, it could be argued that the primary benefit of SLAHMMs might be primarily practical rather than theoretical: they allow the HMM to sample a broader range of sequence space, without the computationally intensive requirement of iterating directly against the entire sequence database.

To help determine the practical vs. theoretical benefits of SLAHMMs, the 42 probes that SLAHMMs uniquely assigned at an EPQ ~0.05 (provided in Table 1) were used as the basis for a smaller-scale test of single-master HMMs vs. SLAHMMs. The superfamilies to which these probes belong were separated, and the single-master HMMs representing them rebuilt. The protocol used (PS1) was identical to that used in the initial experiments, except that in the last iteration, the model was searched against the entire, unfiltered, sequence database ("nr60", see methods), allowing it to potentially discover new homologs previously excluded by the BLAST pre-filter. The new models were then added as a supplement to the model database.

The results of this test suggest that the majority of the benefit of SLAHMMs is theoretical, though there is a substantial practical benefit as well. When given the advantage of a full nr60 search, the single-master HMMs could correctly assign 18 of the probes, but still were unable to assign the remaining 24 (Table 1, column ND2). When given the dual advantages of a full nr60 search *and *accumulation of additional errors out to a theoretical EPQ of 1, the single-master HMMs could still not assign 5 probes correctly assigned by SLAHMMs without these advantages (Table 1, column ND3).

### SLAHMMs provide superior performance for fold-level assignments

The behavior of SLAHMMs suggested that they might show the strongest performance at the very edge of detectable sequence similarity. Therefore, we assessed their performance in assignment of correct *fold level *SCOP similarity. These are cases where the SCOP authors have detected an overall similarity between structures, but there is no current basis to presume an evolutionary relationship [33].

**Figure 4:**
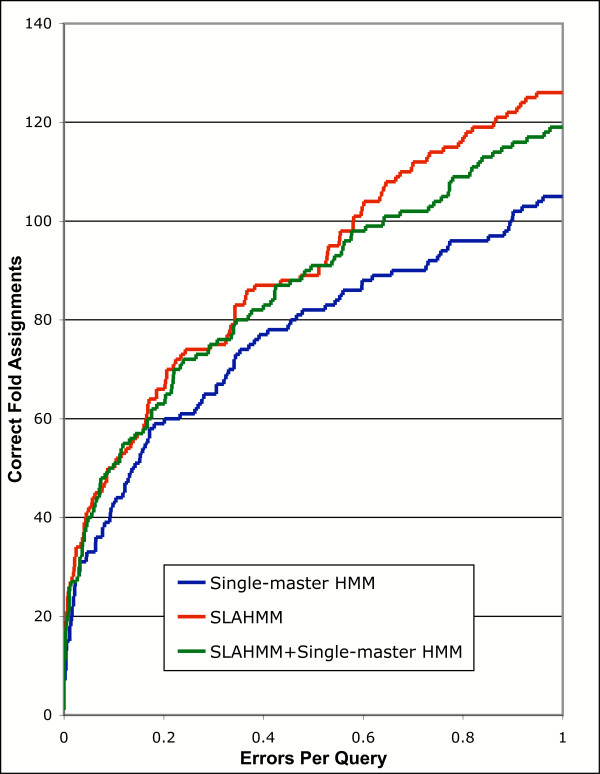
Relative performance of different types of HMMs in assignment of fold-level structure to sequence probes, presented as a coverage vs. error plot. Details of model types are provided in the text. Iterative parameters used for construction of all models were from PS1 (Table 2).

To determine fold assignment performance, correct superfamily-level assignments were ignored, and only cases where a probe was assigned to a *different *superfamily that was in the *same *fold grouping were tabulated. Fold assignment based on sequence is very difficult, and therefore there is a very low success rate [36,37]. However, SLAHMMs substantially outperformed single-master HMMs in fold recognition: at an EPQ ~0.05, SLAHMMs made 42 correct assignments, compared to 33 for single-master HMMs, a ~27% increase (Figure 4). Similarly, at an EPQ of 1 SLAHMMs made 126 correct assignments, compared to 105 for single-master HMMs, a 20% increase. This pattern is the reverse of the results for superfamily-level similarities; it indicates that the methodology used to produce SLAHMMs was successful in generating highly generalized models that capture more distant similarities. However, in this case combining models did not produce any synergy, suggesting that the added noise outweighed any benefit provided to SLAHMMs by the single-master models (Figure 4).

### The benefits of SLAHMMs appear relatively robust to database and software changes

**Figure 5:**
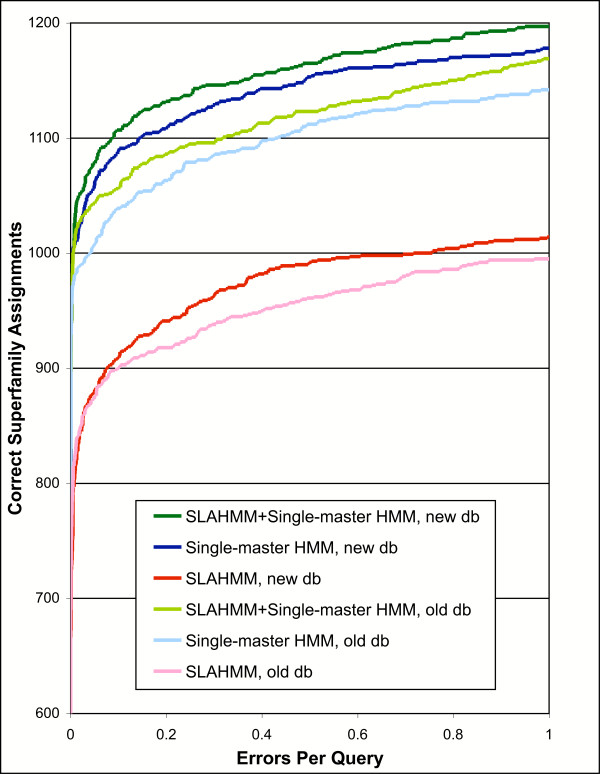
Comparison of HMMs built using an older protein sequence database for iterative construction ("old db") with those built using a current sequence database ("new db"), presented as a coverage vs. error plot. Results are colored similarly for corresponding model types, with the results based on the older database in a lighter color. A different version of the HMMER software was also used for the two result sets; details of model types and construction are provided in the text. Iterative parameters used for construction of all models were from PS1 (Table 2).

Before collection of the final results (presented in Figures 2, 3, 4), we updated the version of HMMER used to build our models, and the version of the sequence database used to collect homologs to our test sequences in our iterative protocol (see methods for details). Apart from providing final results based on the most current data, this allowed us to determine the robustness of our observations in the face of the inevitable growth of sequence databases (and changes to software packages). Direct comparison of the results for PS1 prior to these changes (from Figure 1) and after these changes (from Figure 2) reveal that, while the performance of all methods improved markedly, the essential trends remained in place (Figure 5). These results suggest that the benefits provided by SLAHMMs will continue to be realized, even as sequence databases further expand.

## Conclusion

Our study indicates that structural alignments can be used to improve the results of searches based on profile HMMs. This improvement occurs for the prediction of both superfamily and fold-level relationships. In the case of superfamily-level assignments, SLAHMMs underperformed standard iterated HMMs, but provided a modest improvement in overall performance when used in a combined search, relative to standard HMMs alone. In the case of fold-level assignments, SLAHMMs substantially outperformed standard HMMs. Our experiments with modification of iterative parameters demonstrated that modest changes in the way HMMs are constructed have almost no effect on the performance of the final models. This result suggests that simple adjustments in the construction of sequence-only HMMs will be insufficient to replace the role of SLAHMMs. Further, our work indicates that accurate, structure based alignments are essential for the production of high-quality SLAHMMs.

It has been suggested that the use of superfamily-level structure alignments will lead to "profile dilution", where the additional sequences in the alignment actually reduce the information content, and lead to weaker models [29,32]. However, the success of SLAHMMs in improving the combined search results indicates that, in some superfamilies, there are underlying sequence patterns that are sufficient for the construction of effective models.

This notion is further supported by the strong results of SLAHMMs when tested against fold-level targets from SCOP. Sequences with similarities at the fold level usually do not share any functional motifs and are not presumed to have a homologous relationship, so the sequence match must be made solely based on residue patterns inherent in the production of similar structures. Improvement in HMMs for fold-level structure assignment has been reported through the inclusion of predicted local structure into multi-track HMMs [37]. Our work indicates that SLAHMMs may provide another avenue for the improvement of HMMs for fold recognition.

The computational overhead for the inclusion of SLAHMM-type models into sequence annotation studies is modest, though non-trivial. Multiple structure alignments must be created for the structure representatives in each superfamily. In real-world conditions, only one SLAHMM per superfamily would be required (because structures would not have to be removed for a leave-one-out test, see methods), making the additional HMM training requirements minimal. Since the alignments used to build SLAHMMs are derived from sequence alignments already available from the generation of standard, single-master HMMs, they can be conveniently created as an adjunct set of models to improve overall performance.

The primary limitation of SLAHMMs is that they require at least two structural representatives per superfamily to be modeled, and some superfamilies still lack even a single structural representative. However, with the gradual growth in structural data likely to be generated from the various structural genomics projects [38,39], multiple structure representatives should become available for many more superfamilies. This new structural data will allow SLAHMMs to be used to help provide structural assignments for an increasing percentage of the growing body of genomic sequence.

## Methods

### Sequences/structures used for model building and benchmarking

The SCOP structural classification database [33,34] has become the most common standard used for the benchmarking of protein structure prediction methods [3,11,28,40] and was used in this work. Domains were retrieved from the ASTRAL database (version 1.61 [41,42]). The available ASTRAL pre-filtering was used to collect a set of domains in which no domain could be aligned to another with a BLAST E-value <10^-3^. The intent of the chosen E-value cutoff was to provide a balance between broad coverage of the resulting models (any sequence relationship with a BLAST E-value <10^-3 ^can essentially be considered trivial to detect) and challenging potential assignments with which to test the methods. The chains included the ASTRAL "genetic domains", and were filtered to be at least 80 residues in length. Domains from only the first five classes in the SCOP hierarchy were used, as these represent the "typical" globular proteins to which this work aims to successfully assign structures.

These domains were used both for model building and benchmarking. The filtered ASTRAL set from above was additionally filtered to only retain superfamilies with three or more members, so that models could be constructed based on the structure alignment of two sequences, while still retaining a sequence to test the resulting model (leave-one-out test).

### Building structure alignments for structures in the benchmark set

The selected SCOP/ASTRAL superfamily representatives are distant homologs, and it is difficult to obtain an accurate alignments of them using sequence information alone [23-25]. Therefore, a multiple structure alignment was generated for each superfamily using a variant of the CE software [43] designed to create multiple alignments. Multiple alignments were built through the use of a progressive approach, by using the CE Z-score to generate a guide tree via the UPGMA method [44]. To reduce computational overhead, a single alignment was generated for each superfamily containing all of the domains in the test set for that superfamily. Construction of a single superfamily alignment meant that domains would have to be removed from the structure alignment during benchmarking, as will be described later.

17 structures and one superfamily were removed from the set because of problems caused by inconsistencies between the ASTRAL sequence records and their corresponding PDB files. Also, our CE variant was in still in early development, and failed to align 14 superfamilies. Therefore, these were also removed from the test set. These removals reduced the set of initial structures from 257 superfamilies/1995 chains to 242 superfamiles/1575 chains. However, all five classes of SCOP were still well represented. A breakdown of the representation of SCOP fold classes in this sequence set is avaliable in Additional file 1  of the supplementary material.

### Removing redundancy from the nrprot sequence database

The Genbank non-redundant protein sequence database ("nrprot") was utilized for the collection of sequence homologous to the SCOP domains. Two releases were used, an older release (downloaded 10/18/02) for early development and testing of the method (results shown in Figure 1) and a current release (downloaded 6/9/06) for all final results (Figures 2, 3, 4). A comparison of the results using these two databases is provided in Figure 5. For purposes of homology searches, purging databases of similar sequences does not reduce search performance [45], and in some circumstances may improve performance [46]. The nr databases used in this work were filtered down to a level of redundancy such that no sequence aligned to any other with greater than 60% identity (nr60), using the program cd-hit [47,48].

### Collecting homologous sequence to each SCOP domain with BLAST

The iterative protocol presented here used an initial search with BLAST [17] to capture a set of homologs, which were then aligned with ClustalW [35] to create a seed alignment. The BLAST results, which provide local alignments, were also used to provide the subsection of the sequence homolog that could be aligned to the SCOP domain of interest. Sequences were selected from the BLAST results for inclusion in the ClustalW alignment based on a set of filtration criteria. First, the sequence was required to match the initial SCOP domain with an E-value < 0.001 and sequence identity of >40%. The first measure was used to ensure the hit was likely to be a true homolog; the second was to be sure it could be aligned with high confidence [23,24]. Second, the sequence hit was required to incorporate at least 75% of the SCOP domain sequence in its BLAST alignment. The domains in SCOP generally represent structures that are shared throughout a superfamily, so sequences homologous to a SCOP domain should be able to align to most of it. It was confirmed empirically that aligning all sequences without regard to domain coverage resulted in profile HMMs shortened such that conserved regions at the ends of the SCOP domains were not assigned match states. The coverage requirement does not disallow large insertions in the sequence hit relative to the domain.

The final filtration criterion was that the sequence fragments collected from BLAST could not be more than 90% identical to each other. Because of the domain structure of proteins [49], one domain shared between two proteins can have very high sequence identity, while other domains can still have very low sequence identity. The global filtering done by cd-hit will still retain both of these proteins in nr60, but the domain that is homologous to the SCOP domain could still be highly redundant in the two proteins. Therefore, the sequence fragments collected from BLAST were filtered using the nrdb90.pl program [50].

Each SCOP domain and its homologs were aligned with ClustalW to generate a representative seed alignment of the sequence family. The creation of a seed alignment in this fashion allowed for insertions seen in homologous sequences, but not the SCOP domain, to still be incorporated into the initial model.

The BLAST search results were also used to provide a set of possible homologs for subsequent HMM iteration (described below). All BLAST hits to each SCOP domain with an E-value of < 500 were stored in a miniature database as possible homologs to that domain. HMM searches against the entire nr60 database were prohibitively slow, particularly for purposes of iteration, so pre-filtering the database was required. This technique was first described for the SAM-T98 protocol [18].

### Building profile HMMs representing each of the test domains

The HMMER package [13] was used in this work. Release 2.2g was used for early development of the method (results in Figure 1) and the most recent release (2.3.2) was used for all final results (Figures 2, 3, 4). A comparison of the results for these two versions of HMMER (as well as different versions of the nr database) are provided in Figure 5. HMMER was run with the default settings, except where otherwise noted. The only exception was that 10,000 (rather than the default 5,000) random sequences were used to calibrate the final HMM with *hmmcalibrate*; this provided additional scoring accuracy. Reported E-values were based on the default theoretical background database size in HMMER (59,021 sequences, the size of a version of Swissprot [51]). We found that this setting provided intuitive E-values (i.e. assignments with E >1 were usually incorrect). The bioperl toolkit [52] was used to assist in the collection of outputs from HMMER.

The ClustalW-created sequence alignment was used to train an initial HMM with HMMER. This model was then used to search the pre-filtered sequence database (matching the SCOP domain) for additional homologous sequences. This model was built as a global/local searching model, meaning that it would attempt to capture sequences that span the length of the model. In the first iteration, *all *of the collected sequences were aligned back to the model, including the SCOP domain. The domains collected from BLAST can be somewhat shorter than ideal for representing the sequence space around the SCOP domain (even with filtration for coverage), and the aim of this alignment step was to lengthen the model such that it encompassed all conserved sections of the SCOP domain.

After the first iteration was complete, the alignment produced was again used to build and calibrate another HMM using HMMER. The pre-filtered database was again searched and the sequences scoring below a set E-value threshold (determined by the parameter set as discussed below) were collected and aligned back to the model. The model was then re-built using this new alignment, and another iterative cycle initiated, etc.

We tested 4 different iterative strategies in order to determine which would be the most successful at later structure assignment (Table 2). We name these based on their parameter set (PS). All of the strategies began with a similar seed alignment procedure, but then followed different procedures starting with the first iteration step. First, we will provide a description of the settings used for PS1, then describe the other iterative parameter sets in relation to this baseline.

**Table 2:** Parameters used in tests of different protocols for the iterative generation of HMMs.

**Run Parameter Set (PS)**	**E-value Cutoff Progression (≤)**	**% Coverage Cutoff Progression (>) **	***R***_*im*_**Cutoff Progression (<)**	**Realign All Sequences To Model After Each Iteration?**
1	10^-25^, 10^-10^, 10^-5^, 0.001, 0.01, 0.1	70, 70, 70, 50, 50, 50	10, 3, 3, 3, 3, 3	No
2	10^-25^, 10^-10^, 10^-5^, 0.001, 0.01, 0.1	70, 70, 70, 50, 50, 50	10, 3, 3, 3, 3, 3	Yes
3	10^-6^, 10^-5^, 10^-4^, 0.001, 0.01, 0.1	10 for all iterations	20 for all iterations	No
4	10^-6^, 10^-5^, 10^-4^, 0.001, 0.01, 0.1	10 for all iterations	20 for all iterations	Yes

All runs proceeded through six iterations in order to build an alignment and corresponding profile HMM. Runs are named by their parameter set (PS). For each column describing cutoff values, the values are given in order of iteration cycle, from 1 to 6. In the case of E-value cutoffs, sequences were required to match the model with an E-value less than or equal to the given value. In the case of % coverage, sequences were required to align to a greater percentage of HMM match states than the given value. In the case of *R*_*im*_, (the ratio of inserts to matches, explained in the text) sequences had to score below the given value.

From the second iteration onward in PS1, only the newly collected sequences were freshly aligned to the model, while the prior alignment was retained unchanged. This arrangement allowed sequences at each level of similarity to align to a model most suited to their relationship to the SCOP domain. It also guarded against iteration and alignment drift by anchoring both the alignment and the model to the initial SCOP domain.

The iteration was run for a total of six cycles. With each cycle, the E-value threshold was raised to allow more distant sequences into the alignment as detailed in Table 2.

In addition to the E-value criterion, sequences were filtered prior to addition to the alignment at each iteration based on heuristics that aimed to improve the quality and relevance of the alignments. First, sequences were filtered based on their coverage of the HMM model, calculated as the percentage of match states available in the model aligned to by the sequence. It was determined empirically that a reasonable cutoff level was 70% model coverage in the first three iterations and 50% model coverage in the last three iterations. Sequences that aligned to the model below these cutoffs were almost always short fragments or poor alignments.

In addition to the filtration for coverage, a heuristic was applied to screen out poor alignments. The measure used was the ratio:

Rim=isms
 MathType@MTEF@5@5@+=feaafiart1ev1aaatCvAUfKttLearuWrP9MDH5MBPbIqV92AaeXatLxBI9gBaebbnrfifHhDYfgasaacH8akY=wiFfYdH8Gipec8Eeeu0xXdbba9frFj0=OqFfea0dXdd9vqai=hGuQ8kuc9pgc9s8qqaq=dirpe0xb9q8qiLsFr0=vr0=vr0dc8meaabaqaciaacaGaaeqabaqabeGadaaakeaacqWGsbGudaWgaaWcbaGaemyAaKMaemyBa0gabeaakiabg2da9maalaaabaGaemyAaK2aaSbaaSqaaiabdohaZbqabaaakeaacqWGTbqBdaWgaaWcbaGaem4Camhabeaaaaaaaa@37E1@

where *i*_*s *_is the number of insertion states in the model caused by the sequence and *m*_*s *_is the match states the sequence matched to. The point of this measure was to detect poor alignments in which a sequence only matched a few highly conserved match states, but otherwise aligned to insert states in the model (a high value for *R*_*im*_). These sorts of alignments were sometimes seen at the higher E-value (i.e. lower statistical significance) thresholds, and were presumably not helpful to the construction of a good model. Through the use of *R*_*im*_, large insertions in the model were still possible, provided the sequence also aligned to a large amount of match states. This prevented the exclusion of sequences simply because they produced a large but valid insertion in the model. *R*_*im *_was required to be less than 10 for the initial iteration and less than 3 for all other iterations. *R*_*im *_was set to a very high level in the first iteration because alignments at the low E-value used will almost always provide a very good alignment without any heuristic.

The other parameter sets were arranged to test the validity of the settings used in PS1. PS2 used the same E-value progression, % coverage cutoff progression, and *R*_*im *_progression, but continually re-aligned all sequences back to the new model after every iteration. PS3 and PS4 tested a much less controlled iteration, in which the heuristic parameters were essentially turned off, as well as a more rapid E-value progression (Table 2).

### Storing data on sequences aligned during iteration

As the alignments were iteratively constructed, information about each sequence was stored for later retrieval. The aim of collecting this information was to maintain a record of how well a given sequence matched to a model at the time of its alignment. By extension, this provided information as to which SCOP domain was likely to provide the best alignment partner for a given nr60 sequence. This information was required for the rational combination of the sequence alignments based on the structure alignment of their corresponding SCOP master sequences (described below).

At the end of the iterative cycle each SCOP domain had a matching sequence alignment corresponding to the sequence space surrounding it, as well as a profile HMM that provided a model of the alignment for purposes of sequence searches. At this point, the model was stored as a single-master HMM for later use in the benchmarking experiment, while the sequence alignment was used in the creation of SLAHMMS.

### Using structure alignments to build structure-linked alignment HMMs (SLAHMMs)

Structure alignments were used to generate a high-quality multiple sequence alignment of the SCOP master domains for each superfamily. The sequence alignments to each SCOP master were then grafted on to the structure alignment scaffold by using the one-to-one correspondence of the SCOP master sequence in both alignments. In most cases, the SCOP master did not participate in some columns of the sequence alignment; these columns were removed prior to the merging procedure. In large superfamilies, the resulting alignment could contain thousands of sequences.

The superfamily alignments generated by this merging process often had redundant copies of sequences from the nr60 database. These redundancies occurred because several SCOP masters detected and incorporated the same sequences as they iteratively build alignments. This was expected and desirable, as it meant the HMMs were reaching far into the available sequence space. The redundant sequences were removed such that only the best instance of each sequence was retained.

The best instance of a sequence in an alignment was chosen based on its relationship to the SCOP master that retrieved it during the initial iterative alignment construction. The aim was to select the instance that was likely to be in the highest quality alignment with its SCOP master. This was determined using a set of cascading tests using the data stored during the iteration runs. In order, the tests were: iteration cycle at which the sequence was added to its alignment, the E-value it matched the model with at the time of its addition, and the length of the sequence fragment (a longer length implies a better SCOP master partner for the sequence). The sequence instance which scored best in these tests was retained in the superfamily alignment, and all other instances of the same sequence were removed. Once the redundancy had been removed from the alignment, it was suitable for submission to HMMER to generate a SLAHMM representative of the entire superfamily.

In normal use, it is expected that a single SLAHMM model would be generated and stored. However, for purposes of benchmarking it was necessary to remove a SCOP master and all of the sequence it detected from the alignment, so that that domain could be tested against the (now uncontaminated) model in a leave-one-out test. A version of the alignment for each SCOP master was generated, which was missing that master and its detected sequences. This was done *prior *to the redundancy removal described above, so that the maximum number of sequences collected without the use of the removed domain could be retained. The net effect of the procedure was to create alignments that essentially were constructed as if the removed SCOP master was never present. The SLAHMMs could then be directly compared with the single-master HMMs.

### Difficulties with HMMER when producing SLAHMMs

When producing SLAHMMs with HMMER, difficulties were encountered in a few superfamilies. It appeared that the cause was the input alignments, as these problems were not encountered with the single-master alignments. In some superfamilies, the superfamily-wide alignments were very sparse, with large sections filled mostly with gaps, and areas of conserved structure with little or no recognizable sequence conservation. This type of alignment occasionally caused severe model shortening with HMMER where, despite the input of a large alignment, HMMER would generate a model containing less than 25 match states. In such cases, this problem was addressed by turning off the more sophisticated maximum *a posteriori *method for match state generation [53] and using the simple 50% column representation option to determine model architecture in *hmmbuild*. The use of 25 match states as a cutoff was arbitrary, but seemed reasonable based on reviews of the alignments.

HMMER crashed when attempting to build a model with *hmmbuild *for three alignments in both PS3 and PS4. Therefore, in order to provide PS3 and PS4 with the same amount of models as PS1 and PS2, models from PS1 and PS2 were copied over to their closest partners (PS3 and PS4, respectively) for these cases. In three cases, the models did not make any assignments in the subsequent benchmarking studies. Thus, the added models should have no notable effect on the results for PS3 and PS4. The net result of the above construction steps was 1575 single-master HMMs and 1575 SLAHMMs (built with the superfamily alignments, but each missing a different SCOP master and its corresponding alignment) for each parameter set.

During collection of the final results (Figures 2, 3, 4), HMMER also crashed while attempting to build SLAHMMs for two alignments. These alignments were left out, producing a slight but inconsequential disadvantage for SLAHMMs in that their model set only contained 1573 models.

### Benchmarking standard HMMs against SLAHMMs

The experiments were run by using each SCOP master sequence as a probe against all of the models of each type, as well as a combined database containing all the models. Structure assignments were scored in a "probe-centric" fashion, in which each SCOP domain could only be correctly assigned once. This was done because for each of the 1575 domains, there were at least two correct superfamily-level hits available in the case of single-master HMMs, but only *one *possible correct SLAHMM hit (the SLAHMM that did not consider that domain and its corresponding sequence alignment). Probe-centric scoring allowed for direct comparison of the number of correct assignments between the two methods. All incorrect assignments for a given probe where counted when determining coverage vs. error [11,40], with the exception that each probe could only be incorrectly assigned to a given superfamily once (this was done to suppress possible artifacts resulting from the use of SCOP for benchmarking, see below). Structural assignment of a probe was considered "correct" if the probe was assigned to the correct superfamily as defined by SCOP. Cases where a probe was assigned to the correct fold grouping but not the correct superfamily were ignored (not counted as correct or incorrect).

### Benchmarking standard HMMs against SLAHMMs for fold recognition

Several adjustments were made to the experiment for the benchmarking of fold recognition. Matches of a probe to a model in a *different *superfamily but the *same *fold were counted as correct hits, and matches of a probe to a model in the same superfamily were ignored (this is the opposite of the superfamily benchmarking above, and provided a measure of recognition of sequences with extremely distant similarities). The requirement for a cross-superfamily match meant that some of the probes from the superfamily tests could not possibly be correctly assigned in the experiments (because no models were available from another superfamily in the same fold); these were removed, leaving 675 probes (and possible correct assignments). To expand the size of the test set, we added additional probes from smaller superfamilies which had not been used to make any models because they did not have sufficient structural representatives to meet our initial superfamily filtration criteria. Inclusion of these additional test sequences increased the size of the test set substantially, from 675 sequences from 88 superfamilies to 933 sequences from 291 superfamilies. Thus, the reported theoretical EPQ values for fold recognition (Figure 4) are based on 933 possible correct assignments. For both single-master HMMs and SLAHMMs, all models were used in the benchmarking, including those for which no qualified probe sequence was available. This was done to maintain a comparable "noise background" for the different model sets, which correct matches would have to score above.

### Issues with using the SCOP domain classification

During initial testing, our benchmarking revealed that some aspects of the SCOP classification can lead to misleading results. As we have not seen these issues described in the literature, we provide an overview of them here. A number of probes were assigned with low E-values to structures that were incorrect according to their SCOP classification, but upon further consideration could arguably be seen to be valid profile matches. These "false positive" hits can be caused by two basic problems: possible SCOP underpredictions and HMM artifact generation. The discovery of these problems required both general and specific modifications to the experiment, because incorrect false positives can effectively make more sensitive methods appear to do worse.

### Possible underpredictions in SCOP

Underpredictions refer to cases where the SCOP authors did not group similar structures into the same fold grouping, even though structural comparison indicates a similar overall fold (and perhaps even an evolutionary relationship). Hence, the structures are grouped only into the same *class *in the SCOP hierarchy, a relationship that is not considered to be a correct match for a structure assignment technique. In order to suppress the negative effect of these matches on the results, it was necessary to treat them as fold level matches (i.e. these matches would be ignored in the tabulation of correct and incorrect results).

To determine which fold-level pairings would be provided such special treatment, we adopted a standard based on both sequence and structural characteristics. First, the HMM match between domains at the sequence level was required to be bi-directional (i.e., models from one superfamily were required to detect probes from the other, and *vice versa*). In both directions, an E-value less than 1 was required. Second, the structures providing the sequence match (both from a model and probe standpoint) were required to align with a Z-score of 4 or greater using CE [43]. Structures that can be aligned with a CE Z-score of 3.5 or higher usually represent similar folds. Taken together, these two cutoffs provided a clear demarcation that allowed underpredictions to be counted properly as fold matches for the purposes of the experiment. One exception was made to the Z-score threshold, for the linkage between superfamilies c.30.1 and c.3.1. Structures in these two superfamilies tended to be aligned by CE with Z-scores around 3.5, right at the threshold required to indicate a similar fold. However, the large amount of bi-directional assignments between these two superfamilies with an E-value less than 1 argued strongly for a fold level relationship. Therefore, the two superfamilies were treated as members of the same fold group for purposes of the experiment. A list of possible underpredictions detected is presented in Table 3. Two superfamilies, c.4.1 and c.3.1, had mutual hits that were the result of underprediction in some cases, and artifact generation in others (see below); Table 3 only reports cases that appeared to be caused by underprediction.

**Table 3:** Examples of possible underpredictions detected in the SCOP database.

**Probe Super-family**	**Model Super-family**	**Probe Example**	**Model Example [Built From Domain(s)]**	**Model Type**	***hmmpfam *E-value**	**CE Z-score**	**Total # of Hits**
c.3.1	c.4.1	d1lpfa2	d1lqta2 *et al*. (except m1gtea4)	SLAHMM	1.3 × 10^-5^	4.1	8
c.30.1	c.4.1	d1kjqa2	d1djna3	Single-master	0.1	4.2	4
c.3.1	c.30.1	d1m6ia2	d1iow_1 *et al*. (except d1ehia1)	SLAHMM	0.12	3.7	8
c.66.1	c.3.1	d1inla_	d3grs_2 *et al*. (except d1gpea1)	SLAHMM	0.82	4.2	2 (PS2)
c.66.1	c.30.1	d1jgla_	d1gsoa2	Single-master	0.87	4.4	2 (PS4)
c.78.2	c.30.1	d1b74a1	d1gsoa2 *et al*. (except d1ehia1)	SLAHMM	0.0045	4.1	2

These class-level matches were treated as fold-level matches for purposes of the experiment because of their clear structural and sequence similarity. Although only a one way match is shown in the example cases provided, matches were bi-directional between superfamilies with an E-value of less than 1 in all cases (see text for details). In the case of the single-master HMM examples, the domain used to generate the HMM is provided. For the SLAHMM examples, only the domain used in the CE alignment is provided, although these alignments contain all the domains from the superfamily except one (which is mentioned). In both cases, the CE Z-score reported is for the probe domain aligned to the domain provided with the model. The reported *hmmpfam *E-value is that provided by comparison of the probe with the model. The total number of hits refers to the number of bi-directional hits with E < 1 observed between the given superfamily pair after all possible probe and model combinations. The number provided is from the results of searches with PS1, unless otherwise noted (two relationships were not detected with PS1, but were detected using results from other parameter sets).

All of the possible underpredictions our HMMs detected had some similarity to the "Rossman like folds". Most were α/β/α sandwiches, and all shared a minimal core formed by a parallel β-sheet with a strand order of 2134 (many of the folds had elaborations on this sheet that inserted strands on either or both ends). It seems likely that the regular α/β alternation in these domains produced a distinctive generalized sequence signal that our HMMs detected. It is unclear if these matches were simply the result of analogy or a true homologous relationship. To our knowledge, detectable sequence similarity between these folds has not been previously reported.

### Artifact generation when using SCOP domains

Artifact generation refers to cases where, because of a SCOP domain's architecture, models built using that SCOP domain incorporated sequences related to other SCOP domains. Then, when the experiment was run, these models correctly recognized these other SCOP domains, but this recognition was treated as an "error" because these domains were not part of the same SCOP superfamily as the domain used to initiate the model.

Specifically, such artifact generation occurred where a protein consisted of two interacting fragments that flank a central fragment. In some cases, SCOP split such a protein into two compact domains, with one domain consisting of the central fragment, and the other consisting of the two flanking regions joined together at the break points into a single "composite domain". When this flanking domain was used to build an initial ClustalW alignment in the protocol described above, the central fragment was incorporated via the homologous sequences brought into the initial alignment (it was also possible for the central fragment to be brought into the alignment during the HMM iteration phase). The resulting models then recognized the central fragment as a homolog.

Only two superfamily pairings displayed these types of errors for multiple domain and model matches. In one pair, the domains of b.92.1 (composite domain of metallo-dependent hydrolases) flank those of c.1.9 (metallo-dependent hydrolases). In the other, some (but not all) domains of c.4.1 (nucleotide binding domain) flank those of c.3.1 (FAD/NAD(P)-binding domain). These matches were therefore ignored in the collection of the results.

Cases involving other superfamilies were also detected, but in every one of these cases, the errors did not occur for multiple probes and models across the superfamily. They only occurred for a probe and its corresponding flanking domains with the same PDB ID and chain designation. Therefore, all of these cases could easily be dealt with by adding a simple prohibition to the collection of results: any errors where a probe and matching model shared the both the same PDB ID and chain were ignored. This prohibition automatically removed most artifactual matches, while having a minimal effect on the collection of legitimate errors (identical PDB ID and chain designations are rare for SCOP domains).

### Generalized adjustment of the experiment to help suppress artifacts and underpredictions

Although the methods above removed all obvious cases where correct hits were mislabeled as errors, it was important to institute a generalized method to help suppress the effect of these sorts of errors, in the event that some were missed. This was done by limiting the amount of errors counted for a given probe to one per incorrect superfamily match. This had the effect of allowing artifacts and underpredictions to only add one incorrect match to the total for each probe, as opposed to several. At the same time, it would be expected that limiting counted errors in this fashion would only have a small effect on the counting of legitimate errors, as these sorts of errors tend to be random and so come from multiple different superfamilies for a given probe. As described above, only the first correct match of probe to its superfamily was counted. By only counting each incorrect superfamily once per probe, the experiment effectively asked the question: at a given score cutoff, has this probe been assigned to the correct superfamily, and how many incorrect *superfamilies *scored higher?

## Authors' contributions

ES conceived of the study, performed the experiments and drafted the manuscript. PB helped conceive the study and edited the manuscript. Both authors read and approved the final manuscript.

## Supplementary Material

Additional file 1 Click here for file
