# Supplementary material for: Application of protein structure alignments to iterated hidden Markov model protocols for structure prediction
Source: BMC Bioinformatics. 2006 Sep 14;7:410. doi: 10.1186/1471-2105-7-410 (PMC1622756; doi:10.1186/1471-2105-7-410)
Supplement: Additional file 1 [file 1471-2105-7-410-S1.doc]

**Table S1: Representation of SCOP fold classes in the ASTRAL test set (the ASTRAL class identifier is shown in parenthesis after the class name).**

| **Class** | **Superfamilies** | **Chains** | **Residues** | **Average Chain Length** |
| --- | --- | --- | --- | --- |
| All α (a) | 39 | 279 | 49771 | 178.4 |
| All β (b) | 56 | 348 | 54214 | 155.8 |
| α/β (c) | 68 | 498 | 129019 | 259.1 |
| α+β (d) | 75 | 443 | 73003 | 168.6 |
| Multi-Domain (e) | 4 | 17 | 5368 | 315.7 |
| **All** | **242** | **1575** | **311,375** | **197.7** |
